# Proadrenomedullin in Patients with Preserved Left Ventricular Systolic Function Undergoing Coronary Artery Bypass Grafting

**DOI:** 10.21470/1678-9741-2020-0616

**Published:** 2022

**Authors:** Joanna Stanisz-Kempa, Zbigniew Gąsior, Andrzej Kułach

**Affiliations:** 1 Department of Cardiology, Upper Silesian Medical Center, Katowice, Poland.

**Keywords:** Biomarker, Coronary Artery Bypass, Systolic Function, Proadrenomedullin, ST Elevation Myocardial Infarctation, Perioperative Period

## Abstract

**Introduction:**

A potentially new marker of cardiovascular diseases — proadrenomedullin is the precursor of adrenomedullin, which is a multifunctional peptide hormone, produced in most of the tissues in response to cellular stress, ischemia, and hypoxia.

**Methods:**

Ninety-three people, aged 51-79 years, were included in the study. Exclusion criteria were severe or corrected valvular disease, acute coronary syndrome, age ≥ 80 years, glomerular filtration rate < 45 ml/min, active infectious diseases, and cancer. The subjects were observed for adverse events, including reduced left ventricular ejection fraction (LVEF) by ≥ 10%, first incidence of atrial fibrillation (AF), and the necessity of using dopamine during hospitalization.

**Results:**

Use of pressure amines, occurrence of the first AF episode, and left ventricular dysfunction defined by a decrease in LVEF by at least 10% compared to the value before surgery were reported in the perioperative period. No death, sudden cardiac arrest with effective resuscitation, non-ST-elevation myocardial infarction, ST-elevation myocardial infarction, or heart failure were observed. Significantly higher proadrenomedullin concentration was observed in the group with reduced postoperative LVEF (1.68 *vs*. 0.77 nmol/l, *P*=0.005). The relative risk of a decrease in ejection fraction in the group of patients with proadrenomedullin concentration ≥ 0.77 nmol/l was more than twelve-fold higher (95% confidence interval 1.69-888.33; *P*=0.013) than in the group of patients with a concentration of proadrenomedullin < 0.77 nmol/l.

**Conclusion:**

The higher baseline concentration of proadrenomedullin has a predominantly predictive value of postoperative left ventricular systolic dysfunction.

**Table:** 

Abbreviations, acronyms & symbols			
ADM	= Adrenomedullin	LVEF	= Left ventricular ejection fraction
AF	= Atrial fibrillation	∆ LVEF ≥ 10 points%	= Decrease in LVEF at least 10 percentage points compared to the value before surgery
AUC	= Area under the curve	MR-proADM	= Middle fragment of proadrenomedullin
BMI	= Body mass index	NSTEMI	= Non-ST-elevation myocardial infarction
CABG	= Coronary artery bypass grafting	PAMP	= Proadrenomedullin N-terminal 20 peptide
CAD	= Coronary artery disease	proADM	= Proadrenomedullin
CI	= Confidence interval	ROC	= Receiver operating characteristic
CPB	= Cardiopulmonary bypass	SD	= Standard deviation
DNA	= Deoxyribonucleic acid	STEMI	= ST-elevation myocardial infarction
EuroSCORE	= European System for Cardiac Operative Risk Evaluation	TAVI	= Transcatheter aortic valve implantation
GFR	= Glomerular filtration rate		
LM	= Left main		

## Highlights

Previous studies have shown that elevated level of plasma adrenomedullin reflects the patient's disease, its severity, and prognosis.

Given the above, it was hypothesized that elevated concentrations of proadrenomedullin — precursor of adrenomedullin — can be a predictor of adverse events in the perioperative period. In particular, it was demonstrated that proadrenomedullin is an indicator of a decrease in left ventricular function after coronary bypass grafting in patients with normal systolic function.

## INTRODUCTION

The search for a biochemical marker determining the patient’s prognosis after surgery is justified by the need to constantly improve coronary artery bypass grafting (CABG) results.

**Fig. 1:**
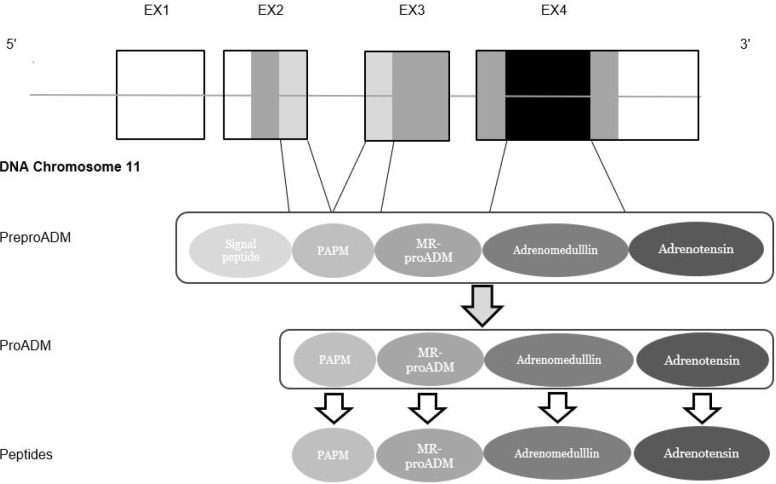
Schematic representation of the proadrenomedullin (proADM) gene^[2]^. DNA=deoxyribonucleic acid; MR-proADM=middle fragment of proadrenomedullin; PAMP=proadrenomedullin N-terminal 20 peptide.

Proadrenomedullin (proADM) is a potential new marker of cardiovascular diseases. The half-life of proADM is several hours, and its plasma concentration proportionally represents levels and activity of adrenomedullin (ADM)^[1]^. ADM is a multifunctional peptide hormone produced in most tissues and many cell types in response to cellular stress, ischemia, and hypoxia. These features indicate that ADM may play a role in protecting against cellular damage and is, therefore, a promising disease biomarker^[2]^. ProADM, an ADM precursor (Figure 1), plays a natriuretic and vasodilatory role and is released in different tissues. It is an autocrine and paracrine mediator, which is an indicator of the severity of a wide spectrum of diseases^[2,3]^. ProADM is a stable active substance and has a longer half-life than ADM^[2]^. The aim of this study was to assess proADM’s predictive value of adverse cardiovascular events in the perioperative period of patients with stable coronary artery disease (CAD) and preserved left ventricular ejection fraction (LVEF) undergoing elective CABG. In addition, the effect of CABG on the dynamics of proADM concentration was assessed.

## METHODS

Ninety-three patients, aged 51-79 years, hospitalized in the Department of Cardiology, and who were qualified for CABG were enrolled. Each patient gave informed written consent to participate in the study.

The study protocol was approved by the Bioethics Committee of the Medical University of Silesia in Katowice (Poland) (Resolution No. KNW/0022/KB1/127/I/13/14 of 07.01.2014).

### Exclusion Criteria

Decreased LVEF (< 50%), acute and chronic inflammatory diseases (within three months preceding), acute coronary syndrome (within three months preceding), age ≥ 80 years old, acute and chronic kidney disease in stages 3b-5 (glomerular filtration rate [GFR] < 45 ml/min/1.73m^2^), malignant tumours, pulmonary hypertension, severe valvular disease, previous CABG, history of valve replacement or repair, autoimmune disease and immunosuppressive treatment, mental and neurological disorders, alcohol abuse, drug use, and anaemia.

### Adverse Events

Patients were observed for adverse events during the perioperative period (hospitalization time from the day of surgery to the day of discharge from Cardiac Surgery Department), which included: death, sudden cardiac arrest with effective resuscitation, myocardial infarction with or without ST-segment elevation, heart failure, the need for pressure amines in the perioperative period, the onset of the first atrial fibrillation (AF) episode, and left ventricular dysfunction defined by a decrease in LVEF at least 10 percentage points compared to the value before surgery (∆ LVEF ≥ 10 points%)

### Laboratory Tests and Echocardiography

Serum morphology, creatinine, and troponin were measured using routine methods in a hospital laboratory before surgery. Troponin was also evaluated on the first day after CABG.

ProADM concentrations were measured by enzyme-linked immunosorbent assay (or ELISA). Measurements were made with one set to avoid variation between tests. ProADM determinations were carried out twice, before surgery and eight weeks after it, at the Department of Pharmacology of the Silesian University of Medicine.

Echocardiographic examination was performed with the use of Vivid E9 (by GE) by one examinator. Left ventricular volume values and LVEF were measured by Simpson’s method at the baseline and before discharge from the Cardiac Surgery Department.

### Statistical Analysis

The analysis of the test results was performed by the IBM Corp. Released 2010, IBM SPSS Statistics for Windows, version 19, Armonk, NY: IBM Corp. and Statistica™, version 10 (2010), programs. The following tests and statistical coefficients were used in the analysis: the Kolmogorov-Smirnov test was used to check the normality of distributions of the analyzed variables; Spearman’s rho correlation coefficient was used to check whether there are statistically significant correlations between quotient variables whose distribution significantly differs from normal; the Mann-Whitney U test was used to check whether there was a statistically significant difference between the two groups in terms of quotient variables whose distribution significantly differs from the normal; and the Kruskal-Wallis test was used to check if there is a statistically significant difference between more than two groups in terms of quotient variables whose distribution is significantly different from normal.

The Chi-squared test was used to check whether there was a statistically significant relationship between the nominal variables. The Wilcoxon rank test was used to check whether there was a statistically significant difference between two parameters of the quotient variable in the same group.

## RESULTS

Characteristics of the studied population are presented in Table 1.

**Table 1:** Characteristics of the study group.

Variable		Value
Male, % (N)		71 (66)
Age, years, mean (SD)		66 (7.5)
Diabetes, % (N)		36.6 (34)
Hypertension, % (N)		100 (93)
Coronary artery disease, % (N)	1 vessel	6.5 (6)
	2 vessels	26.9 (25)
	3 vessels	46.2 (43)
	LM disease	20.4 (19)
Bypass grafts, % (N)	1 graft	9.7 (9)
	2 grafts	50.5 (47)
	3 grafts	37.6 (35)
	4 grafts	2.2 (2)
Smoking history, % (N)		34.4 (32)
BMI, kg/m^2^, mean (SD)		28.9 (4.67)
Atrial fibrillation (paroxysmal or chronic), % (N)		8.6 (8)
ProADM, nmol/l, median (Q1-Q3)		0.91 (0.63-1.52)
EuroSCORE II, %, mean (SD)		1.34 (0.78)
Cardiopulmonary bypass time, min, mean (SD)*		60.2 (18.1)
Aortic clamping time, min, mean (SD)*		34.3 (12.3)
Troponin, ng/ml, median (Q1-Q3)		0.2 (0.135-0.448)
Preoperative LVEF, %, mean (SD)		58.03 (2.53)
Postoperative LVEF, group without decrease in LVEF, %, mean (SD)		55.95 (2.03)
Postoperative LVEF, group with decrease in LVEF, %, mean (SD)		41.9 (5.19)
Postoperative LVEF, total, %, mean (SD)		53.31 (6.21)

* For N=83 (explanation in the text)

BMI=body mass index; EuroSCORE=European System for Cardiac Operative Risk Evaluation; LM=left main; LVEF=left ventricular ejection fraction; N=number of patients; proADM=proadrenomedullin; SD=standard deviation

Out of 93 patients undergoing the procedure, 10 underwent an off-pump surgery. The remaining patients (n = 83) were operated using the traditional method in extracorporeal circulation. The median number of grafts implanted was two.

No statistically significant correlation was observed between age and proADM concentration. Gender, diabetes, smoking history, presence of AF, and stage of CAD also didn’t differentiate patients in terms of proADM levels. Higher levels were observed in people with more advanced CAD (0.74 *vs*. 1.04 nmol/l; *P*=0.054; Mann-Whitney U test) (Table 2)

**Table 2:** Comparison of patients with single and two-vessel CAD with patients with three-vessel CAD and CAD with LM disease in terms of proADM.

Severity of CAD		N	Min.	Max.	Mean	Median	SD	Mann- Whitney U Test
proADM	A	31	0.41	2.94	0.97	0.74	0.62	Z=-1.926
(nmol/l)	B	62	0.33	3.28	1.26	1.04	0.73	P=0.054

A=single or two-vessel CAD; B=three-vessel CAD or CAD with LM disease; CAD=coronary artery disease; LM=left main; N=number of patients; P=test significance; proADM=proadrenomedullin; SD=standard deviation; Z=Mann-Whitney U test statistics

### Adverse Events

The following adverse events have been reported in the perioperative period: use of pressure amines (dopamine) (n = 56), occurrence of the first AF episode (n = 25), and left ventricular dysfunction defined by ∆ LVEF ≥ 10 points% (n = 17) (Table 3). At the same time, two events occurred in 22 patients and three events in five patients.

**Table 3:** Occurrence of adverse events in the perioperative period.

Adverse event	Death	Sudden cardiac arrest with effective resuscitation	STEMI	NSTEMI	Heart failure	Need to use pressure amines in the perioperative period	Occurrence of AF (first episode)	Decrease in LVEF ≥ 10%
Number	0	0	0	0	0	56	25	17

AF=atrial fibrillation; LVEF=left ventricular ejection fraction; NSTEMI=non-ST-elevation myocardial infarction; STEMI=ST-elevation myocardial infarction

After completion of the study, the following parameters were analyzed for the prediction of adverse events: proADM, age, smoking history, diabetes, body mass index (BMI), severity of CAD, European System for Cardiac Operative Risk Evaluation (EuroSCORE) II, aortic occlusion time, cardiopulmonary bypass (CPB) time, and troponin concentration on the first day after surgery.

### Ejection Fraction

**Fig. 2:**
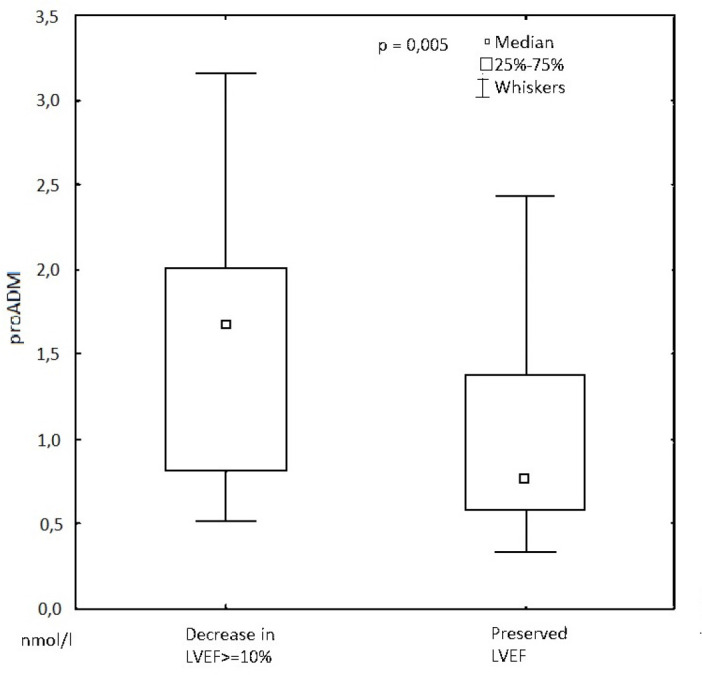
Comparison of patients with a significant decrease in left ventricular ejection fraction (LVEF) ≥ 10% and patients with preserved ejection fraction in proadrenomedullin (proADM) concentration.

No statistically significant impact of age, diabetes, smoking, BMI, or CAD severity on the occurrence of fraction decline was observed. Patients with a significant decrease in LVEF (≥ 10 percentage points) and patients with preserved left ventricular systolic function after CABG did not differ either in EuroSCORE II, CPB time, aortic clamping time, and troponin concentration after surgery. On the other hand, concerning proADM, significantly higher results were observed in patients with reduced left ventricular systolic function after surgery than in those who didn’t have this adverse event (1.68 [0.84-2.0] *vs*. 0.77 [0.62-1.37] nmol/l; *P*=0.005; Mann-Whitney U test). A statistically significant difference is also presented in Figure 2. Among all patients, the lowest mean hemoglobin concentration observed in the postoperative period was 9.81 g/dl (standard deviation [SD] 1.38). In the group of patients with a decrease in the ejection fraction, this value was 9.73 g/dl (SD 1.13). This parameter was comparable among patients in whom this adverse event was not observed (9.83g/dl; SD 1.45). In the group of patients under study, the mean highest concentration of creatinine observed in the postoperative period was 1.047 mg/dl (SD 0.25). There were no statistically significant differences between the group of patients among whom a decrease in the ejection fraction was observed (1.05 mg/dl; SD 0.19) and patients who did not have this complication (1.047 mg/dl; SD 0.27).

### Receiver Operating Characteristic (ROC) Curve

**Fig. 3:**
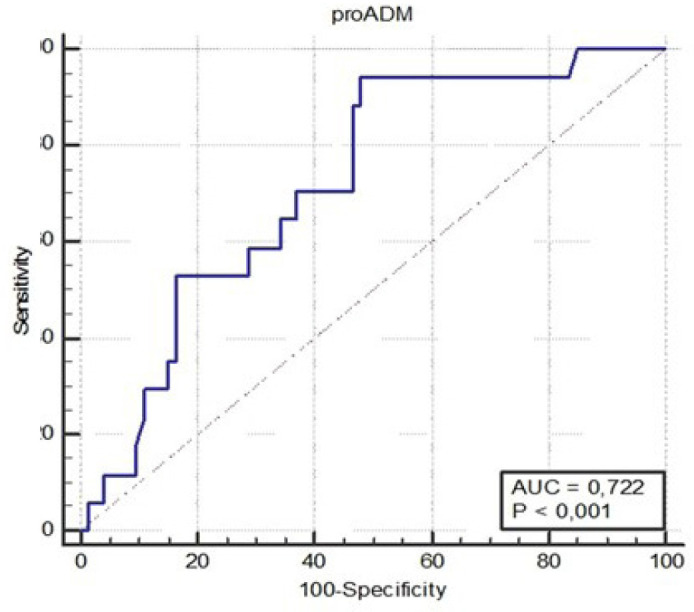
Receiver operating characteristic curve for a decrease in ejection fraction. AUC=area under the curve; proADM=proadrenomedullin.

Analysis of the ROC curve showed that proADM is a pretty good marker of the occurrence of an adverse event, which is a decrease in ejection fraction after CABG (Figure 3). The area under the curve was 0.772, and the optimized cutoff value for proADM was 0.77 nmol/l. This value, with 94.1% sensitivity and 52.1% specificity, shows selected patients who had a complication in the form of a decrease in left ventricular systolic function > 10 percentage points. The relative risk of a decrease in ejection fraction in the group of patients with proADM concentration ≥ 0.77 nmol/l was more than twelve-fold higher (95% confidence interval [CI] 1.69-888.33; *P* = 0.013) than in the group of patients with a concentration of proADM < 0.77 nmol/l. At the same time, the chance (odds ratio) for the occurrence of an adverse event, which is a decrease in ejection fraction in the group of patients with a concentration of proADM > 0.77 nmol/l, was over seventeen times greater than in the group of patients with a lower than the abovementioned proADM concentration (95% CI 2, 19-137.92, *P* = 0.006).

### First AF Episode in the Perioperative Period

Patients who didn’t experience this adverse event did not statistically significantly differ from patients who had an AF episode after surgery in terms of all parameters tested. However, the first episode of AF occurred in a larger percentage of people with diabetes (36.7%) compared to people without diabetes (25.5%) and was also more common in overweight (31.8%) and obese (30%) people compared to people with normal body weight (18.2%). AF was also less common in non-smokers (25.5%) compared to current or past smokers (36.7%). Among patients who had an AF episode after CABG, the median concentration of proADM was 1.02 nmol/l, comparing to the group of patients who did not have this complication (median 0.85 nmol/l).

### Use of Pressure Amines During Hospitalization

A higher percentage of patients who received dopamine were observed among patients with more advanced CAD (62.9% *vs*. 54.8%), with diabetes (67.6% *vs*. 55.9%), and higher body weight (in overweight [58.3%] and obese [69.7%] people, compared to people with normal body weight [41.7%]), as well as in smokers (68.8 % *vs*. 55.7%), although no statistically significant relationship was found between these variables. Higher concentration of proADM have been reported in patients who have used pressure amines (1.12 [0.6-1.8] *vs*. 0.77 [0.5-1.5]; *P*=0.052; Mann-Whitney U test). Troponin levels after surgery were statistically significantly higher (0.23 [0.185-0.45] *vs*. 0.15 [0.11-0.29] ng/ml; *P*=0.004; Mann-Whitney U test).

### Correlation Between Adverse Events and Proadrenomedullin Concentration

The division of patients due to the number of adverse events registered with them allowed to state that the median concentration of proADM is the lowest among 27 people in whom none of the assumed events occurred (median 0.72 nmol/l) and successively increases with the number of recorded events. In the group of patients (n = 39) with one adverse event, the median was 0.85 nmol/l. In case of two events (n = 22), the median increased to 1.06 nmol/L. In five patients, all three adverse events were observed, and, in this case, the median was the highest (1.68 nmol/l; Chi-squared test; Chi-square=9,289; *P*=0,026).

### Dynamics of Proadrenomedullin Concentration

In addition, eight weeks after surgery, the patients’ serum proADM concentration was re-evaluated. It turned out that the concentration of proADM after the procedure was statistically significantly lower compared to baseline (0,71 [0,52-1,15] *vs*. 0,91 [0,63-1,52] ng/ml; *P*<0,001; Wilcoxon test).

## DISCUSSION

It was hypothesized that elevated concentrations of ADM may be a predictor of adverse events in the perioperative period. However, reliable quantification of this peptide is difficult for a short half-life in plasma (~ 22 min)^[2]^, its binding to specific plasma-binding protein, and its physical properties. Most tests cannot detect ADM bound to its binding protein^[4]^. The test performed in this study does not suffer from these restrictions, it assesses the concentration of proADM, the precursor of ADM, and proved to be a tool showing a significantly increased risk of decrease in LVEF after CABG in a cohort of 93 patients.

According to literature data, the most frequently studied biomarker, indirectly correlating with the concentration of ADM, is the middle fragment of proadrenomedullin (MR-proADM). MR-proADM is a stable fragment derived from the same precursor peptide as ADM, releasing in a stoichiometric ratio of 1:1 to ADM^[4]^.

Both MR-proADM and proADM are quite well correlated with ADM plasma levels. However, carried out so far, proADM concentration was explored much less frequently. In this regard, it was decided to analyze the concentration of the precursor molecule for ADM, *i.e*., proADM.

In this study, no correlation was found between the age of patients and the concentration of proADM. SD at level 7.5 indicates a small diversity of the group in terms of age and is the likely cause of no differences in the concentration of proADM according to it. MR-proADM concentration regardless of age was also observed in the von Haehling and Lim studies^[5,6]^. On the other hand, other studies tend to have higher MR-proADM values in older people^[7-9]^.

Gender also does not differentiate proADM levels in this study. A similar relationship has been observed in previous studies in healthy subjects^[6,10]^ and patients with sepsis^[11]^ or with diabetes^[12]^. In the Morgenthaler study of 264 healthy people, MR-proADM showed Gaussian distribution with the mean equal to 0.33 nmol/l^[7]^. There was no difference in this study between the male and female cohort.

Most clinical conditions that could lead to an increase in proADM concentration were excluded from the study. Since everyone involved in the study had adequate blood pressure control as a result of optimal pharmacological treatment, this disease was not included in the analysis.

All recruited patients had CAD qualified for revascularization. It was shown that among patients with one or two-vessel CAD, proADM levels are lower than in the group with three-vessel CAD and left main disease. As far as ADM is known, that increases in CAD and acute coronary syndromes^[13]^, the relationship between the stage of CAD and proADM plasma levels has not been studied yet. In this study, of 93 patients undergoing the procedure, 10 underwent an off-pump surgery. However, this was too few patients to draw meaningful conclusions. Moreover, this subgroup did not differ statistically significantly from the total number of adverse events. The remaining patients (n = 83) were operated using the traditional method in extracorporeal circulation.

No statistically significant differences in proADM concentration were found between the groups of patients with diabetes and patients without this diagnosis. Similar observations were made by Holmanger^[14]^. Diabetes was a predictor of higher MR-proADM concentration in the Leicester Acute Myocardial Infarction Peptide (or LAMP) and Long-term Intervention with Pravastatin in Ischaemic Disease (or LIPID) studies^[8,9]^.

In this study, no differences in proADM concentration were observed between overweight and obese subjects and people with normal body weight. Perhaps this is because only 12.9% of respondents had normal BMI. The remaining patients were overweight or obese, and the average BMI of the test group was 28.9 kg/m^2^. In other studies^[15,16]^, plasma MR-proADM concentrations were significantly higher in obese people than in subjects with normal body weight.

In this study, three adverse events were observed in patients in the perioperative period. In the patients’ group in which there was no decrease in LVEF, the median concentration was over two times lower than in the group in whose fraction decrease was observed. However, these groups did not statistically significantly differ in terms of other parameters tested.

ROC curve analysis showed that a concentration of proADM ≥ 0.77 nmol/l predicts with 94.1% sensitivity the occurrence of a decrease in LVEF ≥ 10 percentage points, and the chance of occurrence of this complication is more than 17 times higher in the group of patients with a higher concentration of proADM.

The sensitivity of our study turned out to be relatively high with the assumed cutoff level, unfortunately with a low specificity of 52.1%. Admittedly, the low specificity of the test will result in too many false warnings. However, the advantage of the test is its high negative predictive value. A positive test result, *i.e*., the determination of an elevated concentration of proADM before CABG (≥ 0.77 nmol/l), could allow for the implementation of early prevention of heart failure and for the abovementioned patients to be subjected to special cardiological supervision.

The reference value of MR-proADM concentration in healthy people according to Gustav-Smith^[17]^ is 0.23-0.64, with a median of 0.41 nmol/l. This result was obtained after examining 1,228 non-smokers, without history of AF, diabetes, stroke, heart attack, hypertension, heart failure, with BMI < 30 and GFR > 60. In this study, the median proADM concentration was 0.91 nmol/l, however, it should be noticed that people qualified for the study were often burdened with several diseases.

In many studies, the prognostic value of ADM has been confirmed in recent years^[5,18-21]^. One of these studies showed that the concentration of MR-proADM in plasma in patients hospitalized for exacerbation of heart failure^[21]^ was almost twice lower in surviving patients (0.79 nmol/l) compared to patients who died (1.37 nmol/l). In the study by Csordas^[3]^, MR-proADM proved to be a predictor of adverse events among patients undergoing transcatheter aortic valve implantation (TAVI). The median concentration of MR-proADM in the studied population of patients with severe aortic stenosis was 1.1 nmol/l, and concentration ≥ 1.3 nmol/l seemed to indicate that the patient is at risk due to no lasting benefit after TAVI.

In the study of Oncel, who examined the concentration of proADM using the kit also used in this study, the cutoff point for patients with sepsis was 3.9 nmol/l, with sensitivity of 86.8% and a negative predictive value of 83.9%^[22]^.

Even though there was no statistically significant relationship between the first incidence of AF and the need for dopamine during hospitalization and the concentration of proADM, the median concentration was lower among patients in whom no adverse event appeared. Furthermore, the concentration of proADM successively increased with the number of recorded events.

Decreased proADM levels after CABG may be the result of improved coronary flow in patients with preoperative normal ejection fraction. The relationship with general hemodynamic stress and endothelial dysfunction of this peptide and its upregulation by hypoxia, cytokines inflammatory, or oxidative stress can help explain its role in the relationship between heart failure and poor prognosis. In turn, improvement in myocardial perfusion after CABG, better oxygenation of cardiomyocytes, and reduction of oxidative stress cause the opposite situation and lower the value of proADM.

### Limitations

A limitation of the study is the relatively small number of patients enrolled, which is largely due to the carefully selected study inclusion criteria.

The limitations related to potential errors in echocardiographic measurements have been minimized by having an experienced echocardiographist performing the examination, using modern ultrasound equipment. The study did not include the assessment of diastolic function — in the population with preserved LVEF, this aspect may be important.

## CONCLUSION

In this study, it turned out that proADM is a new biomarker that can be helpful in predicting adverse events after CABG in patients with preserved LVEF. Higher baseline concentration of this biomarker has a primarily prognostic value of postoperative left ventricular systolic dysfunction. Decreased proADM levels after CABG may be the result of improved coronary flow in patients with preoperative normal ejection fraction.

**Table:** 

Authors' roles & responsibilities	
JSK	Substantial contributions to the conception and design of the work; and the acquisition and analysis of data for the work; drafting the work; final approval of the version to be published
ZG	Substantial contributions to the conception and design of the work; revising the work critically for important intellectual content; final approval of the version to be published
AK	Substantial contributions to the conception and design of the work; and analysis of data for the work; revising the work critically for important intellectual content; final approval of the version to be published
